# The Prohibitin-Binding Compound Fluorizoline Activates the Integrated Stress Response through the eIF2α Kinase HRI

**DOI:** 10.3390/ijms24098064

**Published:** 2023-04-29

**Authors:** Ismael Sánchez-Vera, Sonia Núñez-Vázquez, José Saura-Esteller, Ana M. Cosialls, Judith Heib, Pau Nadal Rodríguez, Ouldouz Ghashghaei, Rodolfo Lavilla, Gabriel Pons, Joan Gil, Daniel Iglesias-Serret

**Affiliations:** 1 Departament de Ciències Fisiològiques, Facultat de Medicina i Ciències de la Salut, Universitat de Barcelona, Oncobell-IDIBELL (Institut d’Investigació Biomèdica de Bellvitge), 08907 L’Hospitalet de Llobregat, Spain; isanchezvera@ub.edu (I.S.-V.);; 2 Laboratory of Medical Chemistry, Faculty of Pharmacy and Food Sciences, Institute of Biomedicine (IBUB), University of Barcelona, 08028 Barcelona, Spain; 3 Departament d’Infermeria Fonamental i Medicoquirúrgica, Facultat de Medicina i Ciències de la Salut, Universitat de Barcelona, 08907 L’Hospitalet de Llobregat, Spain; 4 Facultat de Medicina, Universitat de Vic-Universitat Central de Catalunya (UVic-UCC), 08500 Vic, Spain

**Keywords:** fluorizoline, prohibitin, apoptosis, ER-stress, mitochondrial-stress, ISR, HRI

## Abstract

Fluorizoline is a synthetic molecule that induces apoptosis, by selectively targeting prohibitins (PHBs), through induction of the BH3-only protein NOXA. This induction is transcriptionally regulated by the integrated stress response (ISR)-related transcription factors ATF3 and ATF4. Here, we evaluate the role of the four eIF2α kinases, to decipher which is responsible for the mechanism of ISR activation triggered by fluorizoline in HeLa and HAP1 cells. First, we demonstrated the involvement of the eIF2α kinases using ISR inhibitor (ISRIB) and by simultaneous downregulation of all four eIF2α kinases, as both approaches were able to increase cell resistance to fluorizoline-induced apoptosis. Furthermore, we confirmed that fluorizoline treatment results in endoplasmic reticulum (ER) stress, as evidenced by PERK activation. Despite PERK activation, this kinase was not directly involved in the ISR activation by fluorizoline. In this regard, we found that the eIF2α kinases are capable of compensating for each other’s loss of function. Importantly, we demonstrated that the mitochondrial-stress-related eIF2α kinase HRI mediates ISR activation after fluorizoline treatment.

## 1. Introduction

Fluorizoline is a synthetic molecule that induces apoptosis through selective binding to prohibitin 1 and 2 (PHB1 and PHB2) [1,2]. These proteins are mainly located in the inner mitochondrial membrane (IMM), conforming hetero-oligomeric ring-like complexes. In the IMM, the PHB complex can regulate several processes related to survival, apoptosis, and cell proliferation, among others [3,4].

A growing body of evidence links prohibitins to tumor development. Thus, it has been demonstrated that PHBs mediate cell survival and tumor progression [5,6,7]. Moreover, PHB1 and PHB2 are highly expressed in a wide variety of primary tumors [3], which further supports this pro-tumorigenic role of PHBs. Interestingly, fluorizoline treatment induces apoptosis in a wide range of cancer cell lines [1,2,8,9,10,11,12] and primary samples derived from leukemia and lymphoma patients [13,14,15,16]. Therefore, fluorizoline binding to PHBs clearly inhibits its pro-survival function, emerging as a potential target for cancer treatment.

We have previously described that fluorizoline mainly accumulates in the mitochondria, likely due to its binding to mitochondrial PHBs [1,2]. This binding causes mitochondrial fragmentation, cristae disruption, and reactive oxygen species (ROS) [2,8]. These findings suggest a stressed mitochondrial network, which is compatible with defects in the PHB–mitochondrial complex [17]. Interestingly, one of the best-characterized responses to mitochondrial stress is the mitochondrial unfolded protein response (UPR^mt^), which in turn converges on integrated stress response (ISR) activation [18].

The ISR is a central and evolutionarily conserved adaptive network that is activated in response to various extracellular or intracellular stresses [19]. This pathway has as its central event the phosphorylation of the eukaryotic initiation factor 2 alpha (eIF2α) on Ser51, which leads to general suppression of cap-dependent translation. Thereby, only a subset of mRNAs can be translated in a cap-independent manner, such as the activating transcription factor 4 (ATF4), which can promote pro-survival or pro-apoptotic processes, depending on the stress duration and severity [20,21,22]. Four eIF2α kinases are responsible for the phosphorylation of this factor in response to various stress stimuli: protein kinase double-stranded RNA-dependent (PKR) was initially found to directly react to viral infection, PKR-like ER kinase (PERK) is activated upon ER-stress, haeme-regulated inhibitor (HRI) responds to haeme deprivation, and amino acid deprivation induces the activation of general control non-derepressible-2 (GCN2) kinase. Although each kinase was initially identified as responding to these stimuli, they can also respond to other types of stresses. Moreover, they can eventually cooperate and compensate for the loss of each other [19].

We have recently reported that fluorizoline activates the ISR signaling pathway in different cell lines. On the one hand, we described in HeLa and HAP1 cells that NOXA and apoptosis induction by fluorizoline or PHB downregulation are mediated by the ISR-related transcription factors ATF4 and ATF3 [9]. On the other hand, in HEK293T and U2OS cells, the activation of this pathway showed a protective role upon fluorizoline treatment [8]. Thus, depending on the cellular context, the activation of this pathway by fluorizoline has shown pro-apoptotic or pro-survival roles.

Although PHBs are mainly located in the mitochondria, we have also reported ER-stress induction after fluorizoline treatment in different cell lines, denoted by the splicing of XBP1 through the ER-stress sensor inositol-requiring enzyme 1α (IRE1α) [8]. Disturbances to different ER-related homeostatic processes can impair protein folding efficiency, leading to an accumulation of unfolded or misfolded proteins (so-called ER-stress). This pathway is regulated by PERK, IRE1α, and ATF6 in the ER membrane. Therefore, as ER-stress leads to the activation of PERK, it subsequently triggers the activation of ISR [23].

Therefore, considering that fluorizoline may be triggering both ER and mitochondrial stress, we investigated the role of the four eIF2α kinases, to determine which stress pathway was responsible for the ISR activation in HeLa and HAP1 cells. Here, we describe the role of the ISR in apoptosis execution upon fluorizoline treatment. Moreover, we identify compensatory mechanisms among the four eIF2α kinases, but strikingly the mitochondrial-stress-related kinase HRI emerges as the main driver of the ISR activation in response to fluorizoline treatment.

## 2. Results

### 2.1. ISR Inhibition Increases Cell Resistance to Apoptosis Induction by Fluorizoline

**Figure 1:**
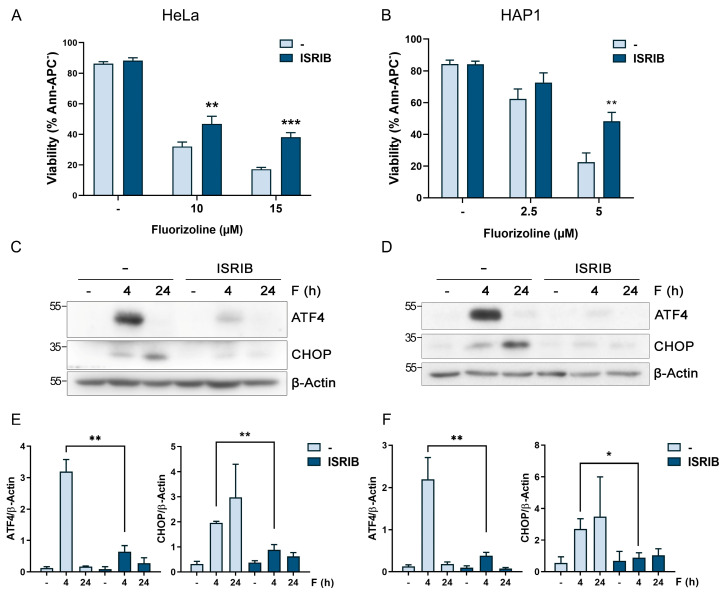
ISR inhibition by ISRIB increases cell resistance to fluorizoline-induced apoptosis. (**A**,**C**,**E**) HeLa and HAP1 (**B**,**D**,**F**) cells were pretreated with 15 µM ISRIB for 1 h. Then, HeLa and HAP1 cells were either untreated (-) or treated with the indicated doses of fluorizoline (F). (**A**,**B**) Cell viability was measured using flow cytometry after 24 h of fluorizoline treatment, and it is expressed as the mean ± SEM (*n* ≥ 3) of the percentage of non-apoptotic cells (Ann-APC^−^). Protein extracts from (**C**) HeLa and (**D**) HAP1 cells either untreated (-) or treated for the indicated times with 10 and 5 µM fluorizoline, respectively, were analyzed with Western blot. β-Actin was used as a loading control. These are representative images of at least three independent experiments. (**E**,**F**) Bars represent the quantification of ATF4 and CHOP relative to β-Actin band intensity. Data show the mean ± SE+M (*n* ≥ 3) of the relative band intensity. * *p* < 0.05, ** *p* < 0.01, *** *p* < 0.001.

We have previously described the relevance of the transcription factor ATF4 for the induction of NOXA and apoptosis after fluorizoline treatment in HeLa and HAP1 cells [9]. Therefore, we first aimed to determine whether ATF4 was increased by the phosphorylation of eIF2α and thus by the activation of any of the eIF2α kinases. In order to investigate the eIF2α kinases’ involvement, we decided to inhibit the pathway at the eIF2α level using the ISR inhibitor (ISRIB), which is capable of avoiding the effect of eIF2α phosphorylation through the activation of the eIF2B complex [19,24]. Importantly, ISRIB pretreatment significantly reduced the induction of apoptosis by fluorizoline in HeLa and HAP1 cells (Figure 1A,B). ISRIB caused a significant inhibition of ATF4 and CHOP induction (Figure 1C–F). This result confirmed that the increase in ATF4 is the consequence of the ISR activation after fluorizoline treatment. Furthermore, these data point towards the involvement of the eIF2α kinases in fluorizoline-induced apoptosis.

### 2.2. PERK Activation by Fluorizoline-Induced Stress Is Not Responsible for the Activation of the ISR

The central event of the ISR is the phosphorylation of eIF2α by any of the four eIF2α kinases. As it has been described that each kinase preferentially reacts to different environmental stresses [25,26], we investigated which kinase is activated in response to fluorizoline treatment. We previously described the presence of ER-stress after fluorizoline treatment in HEK293T and U2OS cell lines [8], and thus the eIF2α kinase PERK emerged as the first candidate to be analyzed.

**Figure 2:**
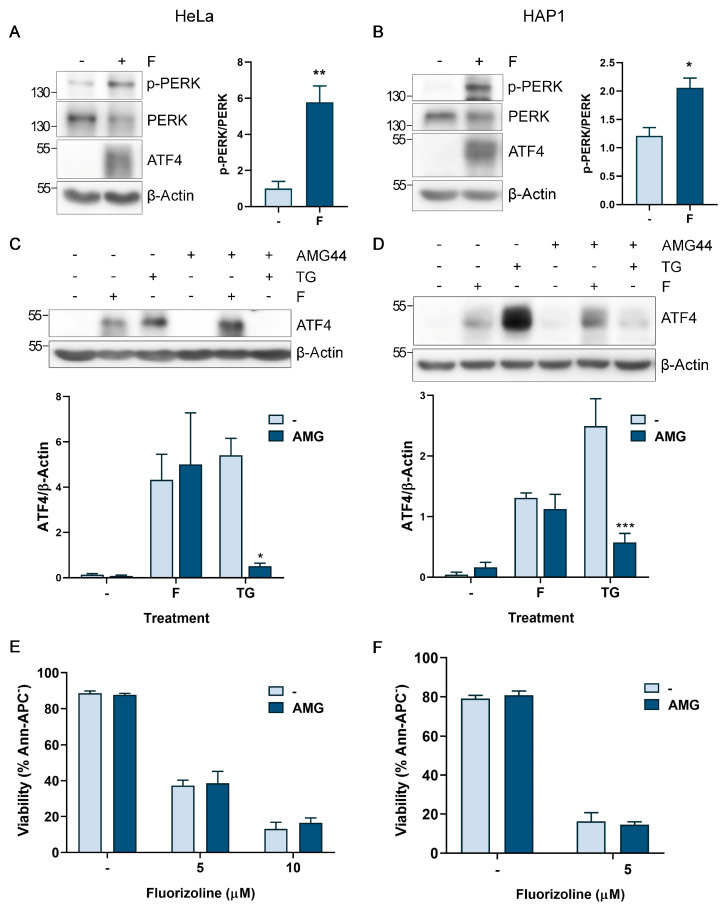
PERK activation is not required for fluorizoline-induced ISR activation and apoptosis. (**A**,**C**,**E**) HeLa and (**B**,**D**,**F**) HAP1 cells were either untreated (-) or pretreated (where indicated) with 2.5 µM AMG44 for 1 h. Then, cells were either untreated (-) or treated with (**A**,**C**) 10 µM fluorizoline (F) or 10 µM thapsigargin (TG) and with (**B**,**D**) 5 µM fluorizoline or 5 µM of thapsigargin. Other doses of fluorizoline are indicated in the figure. (**A**–**D**) Protein extracts from HeLa and HAP1 cells either untreated (-) or treated with fluorizoline for 4 h were analyzed with Western blot. β-Actin was used as a loading control. These are representative images of at least three independent experiments. Bars represent the quantification of (**A**,**B**) p-PERK relative to PERK band intensity and (**C**,**D**) ATF4 relative to β-Actin band intensity. Phosphorylated PERK: p-PERK. Data show the mean ± SEM (*n* = 3) of the relative band intensity. (**E**,**F**) Cell viability was measured using flow cytometry after 24 h of fluorizoline treatment, and it is expressed as the mean ± SEM (*n* = 3) of the percentage of non-apoptotic cells (Ann-APC^−^). * *p* < 0.05, ** *p* < 0.01, *** *p* < 0.001.

To this end, we first analyzed the activation of PERK upon fluorizoline treatment. HeLa and HAP1 cells treated with fluorizoline showed an increase in PERK phosphorylation in Thr982, together with the previously reported ATF4 induction at 4 h (Figure 2A,B). These data demonstrated that fluorizoline treatment results in PERK activation. Therefore, we assessed the involvement of PERK activation after fluorizoline treatment using the PERK inhibitor AMG44 [27]. HeLa and HAP1 cells were pretreated with AMG44 and then, cells were treated with fluorizoline or thapsigargin. As expected, ATF4 induction by the ER stress inducer thapsigargin, which activates the ISR through the eIF2α kinase PERK, was efficiently inhibited upon AMG44 pretreatment (Figure 2C,D). Otherwise, fluorizoline-induced ATF4 protein levels were not affected by the presence of AMG44 in both cell lines, indicating that ISR was still being activated independently of PERK activation (Figure 2C,D). Accordingly, chemical inhibition of PERK did not inhibit fluorizoline-induced apoptosis (Figure 2E,F).

**Figure 3:**
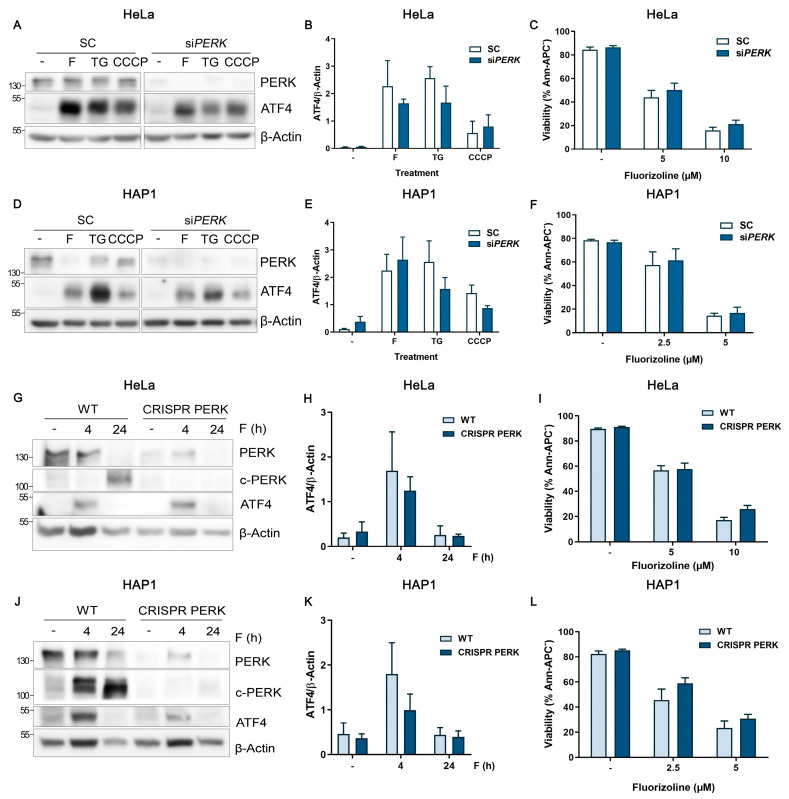
Fluorizoline induces the activation of ISR and apoptosis independently of PERK. (**A**–**C**) HeLa and (**D**–**F**) HAP1 cells were transfected with scramble (SC) or PERK siRNA (siPERK) for 48 h. Then, cells were either untreated (-) or treated with (**A**) 10 µM fluorizoline (F), 10 µM thapsigargin (TG), or 10 µM CCCP; or with (**D**) 5 µM fluorizoline, 5 µM thapsigargin, or 5 µM CCCP for 4 h. (**G**–**I**) HeLa and (**J**–**L**) HAP1 parental cells (WT) and cells lacking PERK (CRISPR PERK) were either untreated (-) or treated with (**G**–**I**) 10 µM or (**J**–**L**) 5 µM fluorizoline, respectively, for the indicated times. Other doses of fluorizoline are indicated in the figure. (**A**,**G**) Protein extracts from HeLa cells either untreated (-) or treated with 10 µM F, TG, and CCCP, and (**D**,**J**) protein extracts from HAP1 cells either untreated (-) or treated with 5 µM of F, TG, and CCCP were analyzed with Western blot for 4 h of treatment or for the indicated times. β-Actin was used as a loading control. Cleaved PERK: c-PERK. These are representative images of at least three independent experiments. (**B**,**E**,**H**,**K**) Bars represent the quantification of ATF4 relative to β-Actin band intensity. Data show the mean ± SEM (*n* = 3) of the relative band intensity. (**C**,**F**,**I**,**L**) Cell viability was measured by flow cytometry after 24 h of treatment, and it is expressed as the mean ± SEM (*n* ≥ 3) of the percentage of non-apoptotic cells (Ann-APC).

We further investigated the PERK involvement in HeLa and HAP1 cells using an acute downregulation approach and also using stable cell lines downregulated for PERK expression. First, PERK was efficiently downregulated using specific siRNA (Figure 3A–F). Afterwards, apoptosis and ISR activation were assessed upon fluorizoline treatment. Additionally, thapsigargin and the mitochondrial stress inductor CCCP were used as controls for PERK and HRI activation, respectively. Importantly, neither ATF4 induction (Figure 3A,B,D,E) nor fluorizoline-induced apoptosis (Figure 3C,F) were affected by the downregulation of PERK in HeLa and HAP1 cells.

Next, we generated pools of HeLa and HAP1 cells downregulated for PERK expression using CRISPR/Cas9 technology (CRISPR PERK) (see Section 4.3). Western blot analysis showed a clear PERK downregulation, although residual PERK protein levels remained, due to the heterogeneity of the cells (Figure 3G,J). WT and CRISPR PERK cells were analyzed for 4 and 24 h after fluorizoline treatment. Treatment with fluorizoline resulted in a significant reduction in overall PERK protein levels. Meanwhile, an increase in a lower band (c-PERK), compatible with that decrease in total PERK, was observed. The downregulation of both bands in CRISPR PERK cells demonstrated that these bands represent specific forms of PERK (Figure 3G,J). This finding was totally reverted upon effector caspases inhibition, indicating that PERK cleavage is produced after effector caspases activation.

According to PERK chemical inhibition, stable downregulation of PERK could not prevent fluorizoline-induced apoptosis in HeLa and HAP1 cells (Figure 3I,L). Indeed, PERK downregulation did not inhibit ATF4 induction by fluorizoline (Figure 3G,H,J,K).

Our results indicate that fluorizoline can still induce the activation of the ISR and apoptosis in the absence of PERK. Altogether, these data indicate that PERK and ER stress are not directly involved in the mechanism of apoptosis induction by fluorizoline.

### 2.3. Analysis of the eIF2α Kinases in Fluorizoline-Induced Apoptosis

Since chemical inhibition and molecular downregulation of PERK did not prevent fluorizoline-induced ISR activation, we next analyzed the other three eIF2α kinases, to identify which kinase mediates the activation of the ISR after fluorizoline treatment.

**Figure 4:**
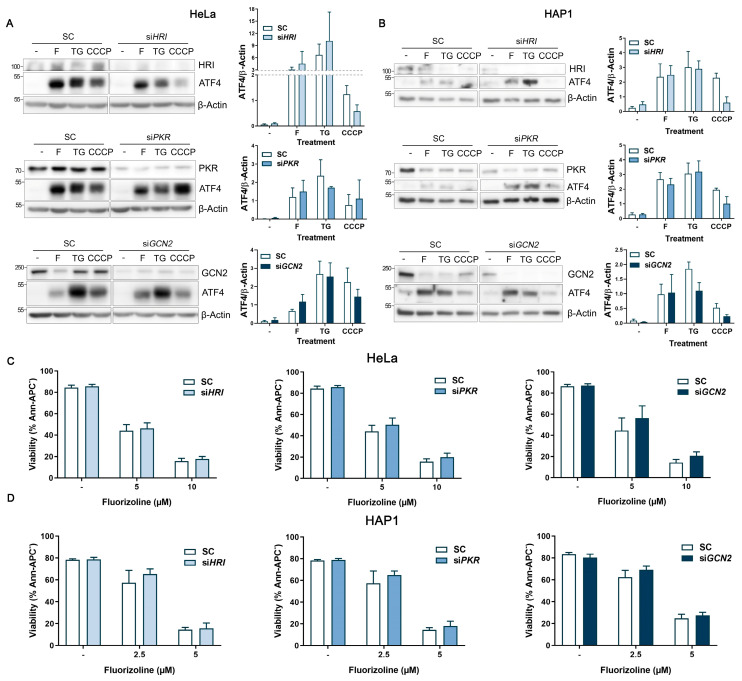
Individual downregulation of HRI, PKR, or GCN2 does not affect the ISR activation and apoptosis induction triggered by fluorizoline treatment. (**A**,**C**) HeLa and (**B**,**D**) HAP1 cells were transfected with scramble (SC) or individually with HRI siRNA (siHRI), PKR siRNA (siPKR), or GCN2 siRNA (siGCN2) for 48 h. Then, cells were either untreated (-) or treated with (**A**) 10 µM fluorizoline (F), 10 µM thapsigargin (TG), or 10 µM CCCP; or with (**B**) 5 µM of fluorizoline, 5 µM thapsigargin or 5 µM CCCP for 4 h. (**A**,**B**) Protein extracts from HeLa and HAP1 cells either untreated (-) or treated (F, TG, CCCP) for 4 h were analyzed with western blot. β-Actin was used as a loading control. These are representative images of at least three independent experiments. Bars represent the quantification of ATF4 relative to β-Actin band intensity. Data show the mean ± SEM (*n* = 3) of the relative band intensity. (**C**,**D**) Cell viability was measured by flow cytometry after 24 h with the indicated doses of fluorizoline, and this is expressed as the mean ± SEM (*n* ≥ 3) of the percentage of non-apoptotic cells (Ann-APC^−^).

HRI, PKR, and GCN2 were efficiently downregulated in HeLa and HAP1 cells (Figure 4A,B). Afterwards, apoptosis and ISR activation were assessed after fluorizoline treatment. Thapsigargin and CCCP were used as ER and mitochondrial stress control treatments. Regarding ISR activation, HRI-downregulated cells showed reduced ATF4 induction upon mitochondrial stress control treatment CCCP, which validated the HRI downregulation (Figure 4A,B). Despite HRI, PKR, and GCN2 downregulation, the ATF4 induction after fluorizoline treatment remained unaffected in both cell lines (Figure 4A,B). Moreover, individual downregulation of the eIF2α kinases did not prevent HeLa and HAP1 cells from undergoing apoptosis upon fluorizoline treatment (Figure 4C,D).

As shown, downregulation of each of the four eIF2α kinases did not show any significant effect on fluorizoline-induced ISR activation, suggesting compensatory mechanisms between the eIF2α kinases upon fluorizoline-induced stress. Indeed, it has been reported that these kinases eventually can cooperate and compensate for the loss of the other eIF2α kinases [25,26]. To test this, we decided to downregulate all four eIF2α kinases simultaneously.

**Figure 5:**
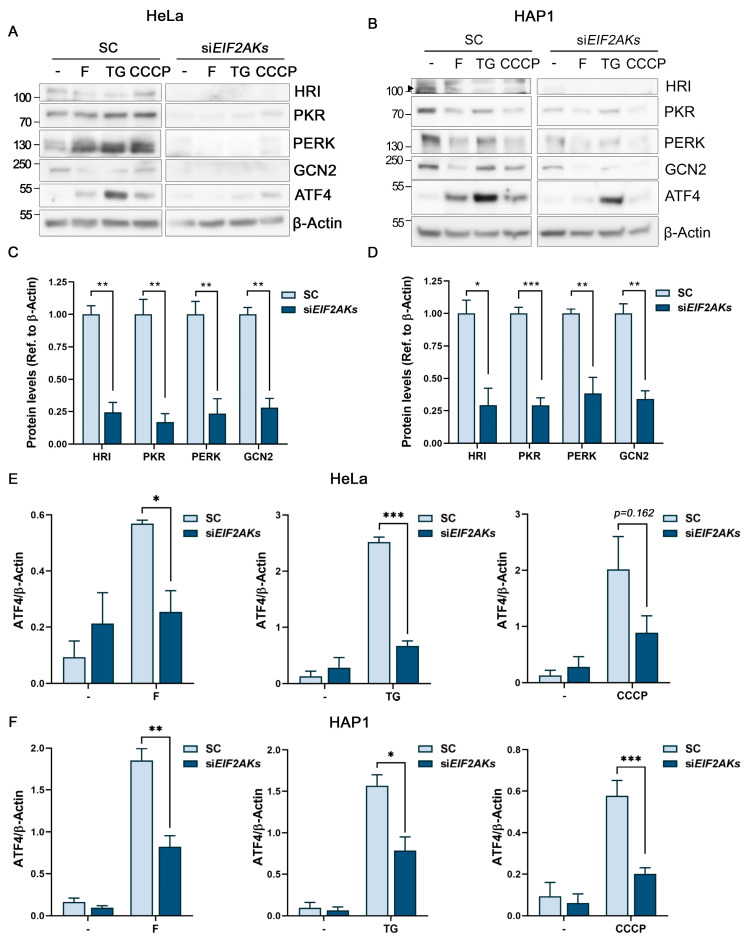
Simultaneous eIF2α kinase downregulation significantly inhibits the ISR activation by fluorizoline. (**A**,**C**,**E**) HeLa and (**B**,**D**,**F**) HAP1 cells were transfected with scramble (SC) or simultaneously with HRI, PKR, PERK, and GCN2 siRNAs (siEIF2AKs) for 48 h. Then, cells were either untreated (-) or treated with (**A**) 10 µM fluorizoline (F), 10 µM thapsigargin (TG), or 10 µM of CCCP; or with (**B**) 5 µM fluorizoline, 5 µM thapsigargin, or 5 µM CCCP for 4 h. (**A**,**B**) Protein extracts from cells were analyzed with Western blot. β-Actin was used as a loading control. These are representative images of at least three independent experiments. Bars represent the quantification of (**C**,**D**) basal levels of HRI, PKR, PERK, and GCN2, and (**E**,**F**) ATF4 inductions relative to β-Actin band intensity. Data show the mean ± SEM (*n* ≥ 3) of the relative band intensity. * *p* < 0.05, ** *p* < 0.01, *** *p* < 0.001.

HeLa and HAP1 cells were efficiently downregulated for each eIF2α kinase (EIF2AK) simultaneously, using specific siRNAs (Figure 5A–D). Moreover, we could validate this approach using the stress inducers thapsigargin and CCCP, as EIF2AK downregulation inhibited ATF4 induction with control treatments (Figure 5A,B,E,F). Interestingly, simultaneous downregulation of the EIF2AKs was able to significantly inhibit the ATF4 induction with 4 h of fluorizoline treatment (Figure 5A,B,E,F), indicating that interfering with the compensatory mechanism among the four eIF2α kinases abrogates the activation of the ISR.

**Figure 6:**
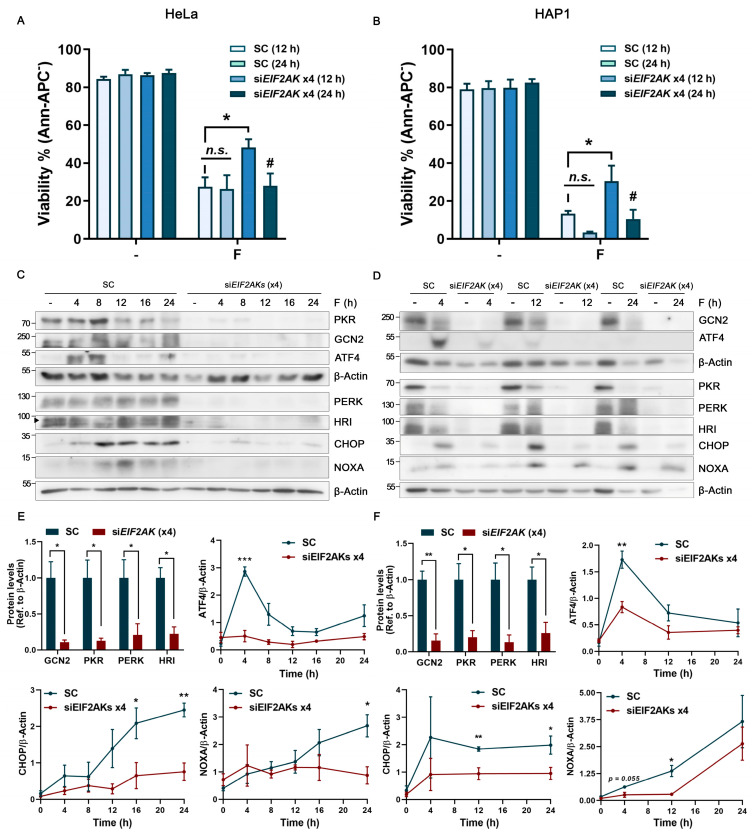
Simultaneous eIF2α kinase downregulation significantly delayed NOXA induction and increased cell resistance to apoptosis after fluorizoline treatment. (**A**,**C**,**E**) HeLa and (**B**,**D**,**F**) HAP1 cells were transfected with scramble (SC) or simultaneously with HRI, PKR, PERK, and GCN2 siRNAs (siEIF2AKs ×4) for 48 h. Then, HeLa and HAP1 cells were either untreated (-) or treated with 10 and 5 µM of fluorizoline (F), respectively, for the indicated times. (**A**,**B**) Cell viability was measured using flow cytometry after 12 and 24 h of treatment, and this is expressed as the mean ± SEM (*n* ≥ 3) of the percentage of non-apoptotic cells (Ann-APC^−^). (**C**,**D**) Protein extracts from HeLa and HAP1 cells were analyzed with Western blot for the indicated times. β-Actin was used as a loading control. These are representative images of at least three independent experiments. (**E**,**F**) Graphs represent the quantification of basal levels of HRI, PKR, PERK, and GCN2, and ATF4, CHOP, and NOXA inductions relative to β-Actin band intensity for all timings analyzed. Data show the mean ± SEM (*n* ≥ 3) of the relative band intensity. * *p* < 0.05, ** *p* < 0.01, *** *p* < 0.001, not statistically significant: *n.s.*, SC versus siEIF2AKs ×4 transfected cells; # *p* < 0.05 12 h versus 24 h of fluorizoline treatment.

Next, we investigated the relevance of the eIF2α kinases in the apoptotic execution triggered by fluorizoline. Interestingly, the HeLa and HAP1 cells simultaneously downregulated for the four eIF2α kinases showed significant resistance to fluorizoline-induced apoptosis at 12 h, although these differences decreased at 24 h (Figure 6A,B). Of note, fluorizoline-induced apoptosis did not increase significantly from 12 to 24 h in the control group, whereas in the EIF2AKs downregulated cells, apoptotic cell death significantly increased at the latest time-point of 24 h (Figure 6A,B). This result indicates that the ISR inhibition through EIF2AKs downregulation delayed the apoptosis execution in HeLa and HAP1 cells.

As stated above, we recently reported that the induction of the pro-apoptotic protein NOXA after fluorizoline treatment was mainly mediated by ATF4 in HeLa and HAP1 cells [9]. Therefore, we analyzed ATF4 and its downstream factors CHOP and NOXA after fluorizoline treatment (Figure 6C,D). In concordance with the viability profile, EIF2AKs-downregulated cells showed a significant inhibition of the ATF4 induction peak at 4 h after fluorizoline treatment. Similarly, a significant inhibition of CHOP induction over time was observed. NOXA protein levels remained significantly inhibited until the 12 h time point in both cell lines analyzed. In particular, in HeLa cells, NOXA inductions were completely inhibited throughout the entire time course, whereas in HAP1 cells, NOXA induction was restored at 24 h of fluorizoline treatment (Figure 6C–F).

All these data indicate that the inhibition of the ISR at the EIF2AK level significantly reduces ATF4 induction, being less efficient in inducing CHOP, NOXA, and apoptosis.

This result further demonstrated that ATF4 induction by fluorizoline can be carried out by any of the eIF2α kinases through the activation of the ISR. Furthermore, it supports the hypothesis that more than one of the eIF2α kinases, which have overlapping functions, could play a role in the mechanism of action of fluorizoline, perhaps due to cooperation among them or through compensatory mechanisms.

### 2.4. Fluorizoline Treatment Activates the eIF2α Kinase HRI to Induce the ISR

**Figure 7:**
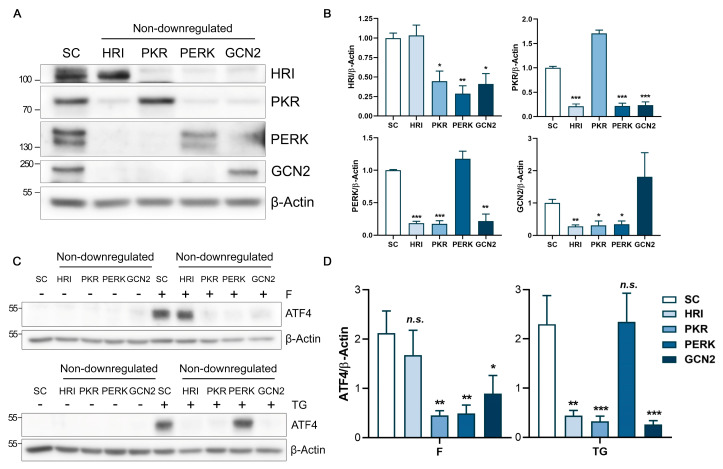
Fluorizoline primarily induces the activation of the ISR through HRI. (**A**–**D**) HeLa cells were transfected with scramble (SC) or simultaneously with a triple combination of the EIF2AKs siRNAs for 48 h, allowing the endogenous expression of one of them. Each combination is defined by the kinase that was not downregulated: HRI, PKR, PERK, or GCN2. Then, cells were untreated (-) or treated with (**C**) 10 μM fluorizoline (F) and 10 μM thapsigargin (TG) for 4 h. Protein levels were analyzed with western blot. β-Actin was used as a loading control. These are representative images of three independent experiments. Bars represent the quantification of (**B**) HRI, PKR, PERK, or GCN2 and (**D**) treatment-induced ATF4 relative to β-Actin band intensity. Data show the mean ± SEM (*n* = 3) of the relative band intensity. Statistically significant reductions: * *p* < 0.05, ** *p* < 0.01, *** *p* < 0.001, not statistically significant: *n.s.*

To overcome potential compensatory mechanisms among the eIF2α kinases, we designed an approach to analyze the role of each kinase in the fluorizoline-induced ISR, without the overlapping effects of the other kinases. As it was essential to achieve high downregulation efficiencies, HeLa cells were selected for this approach. To this end, we simultaneously downregulated three of the four eIF2α kinases, allowing one of them to be physiologically expressed. Then, cells were treated with fluorizoline or with thapsigargin for 4 h. High downregulation efficiency was obtained in each interference combination (Figure 7A,B). This experiment was validated using thapsigargin as a PERK-dependent control treatment, which showed that cells exclusively expressing PERK could induce ATF4 to the same extent as the control cells (Figure 7C,D). Interestingly, we found that after fluorizoline treatment where HRI was the unique non-downregulated eIF2α kinase, ATF4 protein levels were induced to the same extent as the control cells (Figure 7C,D). These results demonstrated that HRI is the main kinase that senses fluorizoline-induced stress to trigger the activation of the ISR.

## 3. Discussion

In this article, we investigated the involvement of the eIF2α kinases in the activation of the ISR, and their role in the mechanism of apoptosis induction by fluorizoline. Our findings demonstrate that fluorizoline-induced apoptosis is attenuated or delayed when the ISR activation is compromised at the eIF2α kinase level. Importantly, we demonstrated that fluorizoline mainly triggers the activation of the ISR through the eIF2α kinase HRI.

**Figure 8:**
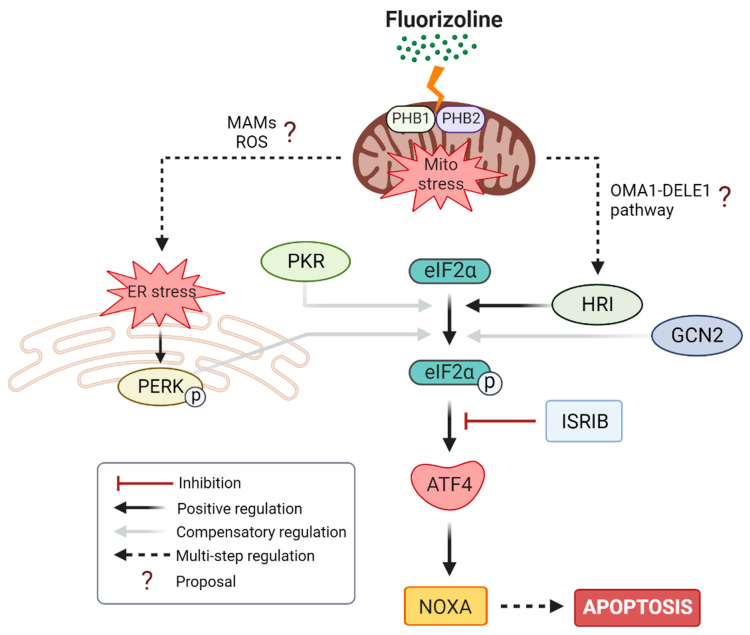
Schematic representation of the proposed fluorizoline mechanism of action. Fluorizoline molecules bind to PHB1 and PHB2 in the inner membrane of mitochondria, disrupting their normal functions and leading to mitochondrial stress. Upon this stress, the ISR pathway is mainly activated through the eIF2α kinase HRI, perhaps through the OMA1-DELE1-HRI pathway. Compensatory mechanisms within all four eIF2α kinases were found. Alternatively, fluorizoline treatment resulted in ER stress and PERK activation, perhaps due to the close relation of mitochondria and ER in MAMs or as a consequence of fluorizoline-induced ROS. Finally, the activation of this pathway converged in ATF4-induced NOXA protein upregulation and apoptosis execution.

Previous results have already pointed out that fluorizoline-induced ER stress could be the cause of the activation of the ISR. We could identify the activation of IRE1α through the splicing of XBP1 in HEK293T and U2OS cells [8]. Moreover, other studies also related the activation of the ISR after fluorizoline treatment to ER stress [10]. In this work, we confirmed the presence of ER stress, by identifying PERK activation in response to fluorizoline treatment in HeLa and HAP1 cells. Although all these data indicated that the activation of the ISR may be due to ER stress, PERK chemical inhibition and molecular downregulation had no effect in the activation of the ISR and apoptosis execution. Therefore, ER stress is not the initial source of the stress induced by fluorizoline and responsible for triggering the ISR activation and the apoptotic outcome. However, we cannot discard PERK involvement at a later stage. The mechanism by which fluorizoline binding to PHBs induces ER stress remains to be elucidated. PHBs are not located in the ER, but there is growing and consistent evidence describing mitochondria and ER associations, mitochondrial-associated membranes (MAMs), as key regulators of calcium homeostasis, proteostasis, mitochondrial bioenergetics, apoptosis, and autophagy [28]. Furthermore, the presence of PHB at MAMs fractions has been observed [29]. In this regard, we reported Ca^2+^ mobilization after treating U2OS cells with fluorizoline [8], which was also observed in A549 cells by other researchers [10]. Interestingly, PHBs have been reported to interact with the voltage-dependent anion channel (VDAC) in the outer mitochondrial membrane (OMM). VDAC is involved in Ca^2+^ signaling from the ER to the mitochondria at MAMs, through its interaction with the mitochondrial calcium uniporter (MCU) in IMM and with IP_3_ receptor at the ER membrane [28]. Thus, fluorizoline may impair ER-Ca^2+^ homeostasis. Furthermore, a direct localization of PERK in MAMs has been found. Indeed, PERK-depleted cells showed a decrease in ER-mitochondria contact sites and an increased apoptosis resistance against agents that induce ER stress through ROS production [30]. These data, together with previous results describing ROS production after fluorizoline treatment [2,8] might explain the activation of PERK (Figure 8).

In this study, we found that individual downregulation of the eIF2α kinases failed to identify the involvement of any of the four kinases in ATF4 induction by fluorizoline, suggesting compensatory mechanisms between them. This result is consistent with previous data describing ATF4 as a key regulator of the mitochondrial stress response, whereby individual eIF2α kinases downregulation did not abolish ATF4 induction [31]. In addition, the general activation of all four eIF2α kinases upon stress has been reported [21]. For instance, compensatory functions between PERK and GCN2 kinases have also been described in mouse models and HeLa cells [26,32,33].

We demonstrated that the ISR activation by the eIF2α kinases participates in the apoptotic induction upon fluorizoline treatment in HeLa and HAP1 cells, as the inhibition of the pathway by ISRIB could decrease fluorizoline-induced apoptosis. Interestingly, we demonstrated that the activation of the ISR was driven by one of the eIF2α kinases, as the simultaneous downregulation of all four kinases significantly inhibited the ATF4 induction by fluorizoline. Furthermore, this interference significantly increased the cell resistance to fluorizoline-induced apoptosis in HeLa and HAP1 cells at 12 h, which was reduced at the longer time point of 24 h. Accordingly, NOXA inductions were inhibited, especially up to the 12 h time point. However, NOXA induction was restored at the latest time point in HAP1 cells, which may indicate the activation of an ISR-independent mechanism of NOXA induction. These data suggest that fluorizoline induces an acute overactivation of the ISR, to induce apoptosis. Moreover, our findings indicate that when the ISR activation is compromised, either by ISRIB or by EIF2AK downregulation, the apoptotic outcome is delayed. Finally, as the fluorizoline-induced stress is sustained over time, the attenuated ISR activation could be sufficient to eventually induce the apoptotic outcome.

To overcome these compensatory mechanisms of each eIF2α kinase, we performed an approach that allowed us to analyze the eIF2α kinase involvement, while avoiding their overlapping activities upon fluorizoline treatment. Strikingly, by using this approach, we could identify HRI as the kinase responsible for the activation of the ISR upon fluorizoline treatment, likely due to the mitochondrial stress induced by fluorizoline (Figure 8). The eIF2α kinase analysis here described upon fluorizoline treatment is consistent with other previous data indicating different compensatory mechanisms for sensing mitochondrial stress within the cell [34]. These data may rule out the notion of a unique pathway relating mitochondrial stress to the activation of the ISR. Instead, it seems that distinct molecular mechanisms are able to sense this stress, depending on the cellular context.

Recently, a molecular pathway linking mitochondrial stress to the activation of HRI has been described. This pathway is coordinated by the IMM protease OMA1, which proteolyzes the intermembrane space (IMS) small protein DELE1 to activate HRI [35,36]. Therefore, this pathway is compatible with the HRI involvement in the ISR activation triggered by fluorizoline described here. Moreover, several studies have also related mitochondrial dysfunction, such as electron transport system (ETS) inhibition or mitochondrial ROS to GCN2 activation [34,37]. Therefore, it is plausible that the initial stress induced by the fluorizoline binding to PHBs at the mitochondria triggers a molecular pathway leading to the activation of cytosolic eIF2α kinases, and thus GCN2 could compensate for the loss of HRI. Interestingly, it has been pointed out that ATF4 can still be induced in HRI or DELE1 downregulated cells with a time delay [35], further suggesting the notion of these compensatory activities among the eIF2α kinases.

Mitochondrial PHB complexes are closely related to mitochondrial quality control processes such as OXPHOS assembly, ROS formation, mitochondrial DNA organization, and mitophagy [4,38]. Furthermore, we and others have found an activation of the ISR upon PHB depletion in human cancer cells and in *C. elegans* [8,9,39]. According to these PHB functions, targeting PHBs with fluorizoline treatment led to mitochondrial fragmentation, cristae disorganization, and mitochondrial ROS production; a clear mitochondrially stressed phenotype compatible with the PHB-downregulated cells [1,40]. Moreover, mitochondrial PHBs complexes serve as scaffolds for the reciprocal stabilization of some proteins, including SLP2 and, interestingly, OMA1 [41]. As a result of this interaction between PHBs and OMA1, it has been reported that downregulation of PHBs can lead to the stabilization of the OMA1 active form [42,43]. Accordingly, a recent study related the activation of the ISR to mitochondrial stress through the activation of OMA1 triggered by the disruption of the interaction between PHBs and SLP2 [44]. All these data further reinforce that fluorizoline could be inducing HRI activation through the OMA1-DELE1-HRI pathway.

Here, we describe that the inhibition of the ISR in HeLa and HAP1 cells increased cell resistance to fluorizoline-induced apoptosis. Furthermore, we previously described that, in HEK293T and U2OS cells, inhibition of the ISR caused an increased sensitization to fluorizoline-induced apoptosis [8]. These data support the notion that the different basal stress states that are present in human cancer cells from different origins may lead to distinct responses to treatments that induce ISR activation. Therefore, either reducing or increasing ISR activation can impair cancer cell survival upon a stressful stimulus. For instance, in cancer treatment, some approaches have focused on overloading the stress that a cell can handle [45,46,47]. Conversely, upregulation of the ISR has been related to the development of drug resistance in cancer [22,48]. Hence, opposite approaches have been developed to inhibit some components of the ISR, to overcome such resistance, such as eIF2α kinases inhibitors [49,50,51,52], and eIF2B activators, such as ISRIB and 2BAct [53,54]. Therefore, further efforts should be made to better understand the regulation of the ISR in cancer, in order to adequately modulate the ISR, as a promising targeting pathway for cancer treatment [55].

In conclusion, in this article, we characterized the fluorizoline-induced stress response and apoptosis in HeLa and HAP1 cells. Interestingly, the apoptosis induced by fluorizoline was found to be the result of a potent activation of the ISR mediated by HRI, likely due to mitochondrial stress. These results improve our understanding of the cell response to treatment with the PHB-binding compound fluorizoline, which in turn could be valuable in developing future therapeutic applications targeting PHBs in cancer treatment.

## 4. Materials and Methods

### 4.1. Cell Lines

A HeLa cell line, derived from cervical cancer cells, was supplied by the European Collection of Authenticated Cell Cultures (ECACC). HAP1 cells, a near-haploid human cell line that was derived from the male chronic myelogenous leukemia (CML) cell line KBM-7 were by supplied Horizon Discovery. HeLa cells were cultured in Dulbecco’s Modified Eagle Medium (DMEM) and HAP1 cells were cultured in Iscove’s Modified Dulbecco’s Medium (IMDM). DMEM and IMDM were supplemented with 10% fetal bovine serum (FBS) and heat-inactivated FBS (FBSi), respectively, and 100 ng/mL gentamycin. DMEM was also supplemented with 2 mM L-glutamine (all from Biological Industries, Israel). Cells were cultured at 37 °C in a humidified atmosphere containing 5% carbon dioxide.

### 4.2. Reagents

The synthesis of fluorizoline was performed as previously described (Pérez-Perarnau et al., 2014b). DMSO, ISRIB, thapsigargin, and CCCP were supplied by Sigma-Aldrich (Merck, Saint Louis, MI, USA).

### 4.3. CRISPR/Cas9

To generate a pool of HeLa and HAP1 cells lacking a specific protein, we used the system previously described in [56]. Different short guide RNAs (sgRNA) were designed to target PERK (5′-AAACAGACCGTGAAAGCATGGAAAC-3′). The sgRNA sequences were cloned into the pSpCas9(BB)-2A-puro vector (supplied by Adgene, Watertown, MA, USA), which encodes an RNA Polymerase III promotor for the transcription of the guide, the Cas9 endonuclease and a gene providing resistance to puromycin. HeLa cells were transfected overnight with Lipofectamine^®^ LTX Reagent (Thermo Fisher Scientific, Waltham MA, USA) and HAP1 cells with Turbofectin from Origene (Rockville, MD, USA). An empty vector without sgRNA was used as a negative control. Puromycin was added for 24 h at 2 and 1 μg/mL for HeLa and HAP1 cells, respectively, to select PERK-depleted cells. The selected cells were tested for gene deletion using a mismatch cleavage assay and checked for protein knock-down with an immunoblot. Only those cell cultures showing clear PERK downregulation were used for experiments.

### 4.4. Cell Viability

Cell viability was assessed by measuring phosphatidylserine exposure with annexin V APC staining and analyzed with flow cytometry using FACSCantoTM and FACSDivaTM software 6.1.3 (Becton Dickinson, NJ, USA). Cells were incubated with annexin binding buffer and annexin V-APC for 15 min in the dark before analysis. Cell viability was expressed as the percentage of the annexin V-APC-negative population, which corresponds to the non-apoptotic cells.

### 4.5. Small Interfering RNA Transfection

HeLa cells were transfected with siRNA of *EIF2AK1* (HRI, S25800), *EIF2AK2* (PKR, S11185), *EIF2AK3* (PERK, S18102), *EIF2AK4* (GCN2, S532694), and their negative control (4404021) using Lipofectamine^®^ RNAiMax Reagent (all purchased from Thermo Fisher Scientific, Waltham, MA, USA). DMEM and IMDM were replaced with OptiMEM (Gibco, ThermoFisher), and complexes were added into cells dropwise and incubated for 4–6 h and then replaced again with their respective fresh media. The efficiency of the downregulation was assessed through Western blot.

### 4.6. Western Blot

Whole-cell protein extracts were obtained by lysing cells with Laemmli sample buffer. Protein concentration was measured with a Micro BCA Protein Assay Reagent kit (Pierce, Rockford, IL, USA). Protein extracts (20–40 µg) were subjected to reducing conditions, loaded onto a polyacrylamide gel, and then transferred to Immobilon-P membranes from Millipore (Billerica, MA, USA). One hour after blocking with 5% (*w*/*v*) non-fatty milk in Tris-buffered saline solution with Tween^®^ 20, membranes were incubated with the following specific primary antibodies: β-Actin (AC-15, Sigma-Aldrich), ATF4 (D4B8, Cell Signaling), CHOP (2895, Cell Signaling), NOXA (114C307, Abcam), HRI (MBS2538144, MyBioSource), PKR (3072S, Cell Signaling), PERK (C33E10, Cell Signaling), p-PERK (Thr982) (PA5-102853, Invitrogen), and GCN2 (3302S, Cell Signaling). Antibody binding was detected using a secondary antibody conjugated to horseradish peroxidase, and an enhanced chemiluminescence detection system (Amersham, Little Chalfont, UK). Raw quantification of band intensities was performed using Multi Gauge software V3.0 (Fujifilm Corporation).

### 4.7. Statistical Analysis

The results are shown as the mean ± standard error of the mean (SEM) of values obtained in three or more independent experiments. Statistical analysis was performed using Student’s *t*-test (two-tailed) for simple comparisons or ANOVA–Tukey for multiple comparisons, using GraphPad Prism 6.0c Software Inc. Differences were considered significant at *p* values below 0.05 (* *p* < 0.05; ** *p* < 0.01; *** *p* < 0.001).

## Data Availability

Not applicable.
